# Supplementary figures and images for: Differences in structure and hibernation mechanism highlight diversification of the microsporidian ribosome
Source: PLoS Biol. 2020 Oct 30;18(10):e3000958. doi: 10.1371/journal.pbio.3000958 (PMC7644102; doi:10.1371/journal.pbio.3000958)

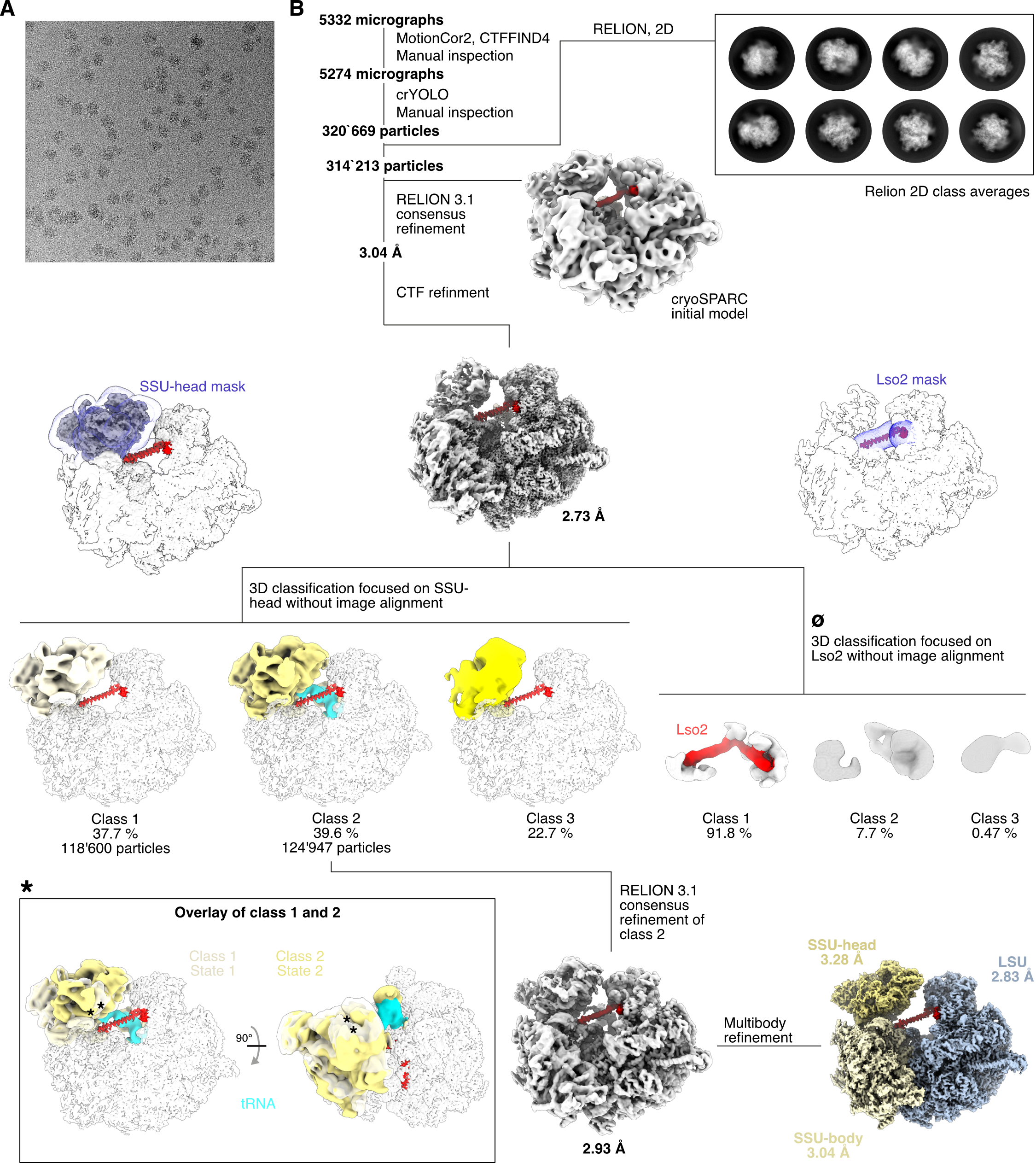

Supplement: S1 Fig — (A) Representative cryo-EM micrograph of the microsporidian ribosome. (B) The 5,332 collected micrographs were manually inspected to remove those with drift, poor CTF fits, or low-quality ice, resulting in a total of 5,274 micrographs. Particles were picked using crYOLO [39] and subjected to 1 round of 2D classification (representative 2D class averages shown) in RELION-3.1 [41] to remove picking contaminations. The initial model was generated using cryoSPARC [40]. A consensus refinement yielded a map at 3.0-Å resolution, which was improved further by per-particle CTF refinement to a resolution of 2.73 Å. To isolate the most populated conformation of the dynamic SSU-head region, a focused 3D classification was performed without image alignment. The resulting 3 classes of the SSU-head domain (different shades of yellow) are shown superimposed with the full consensus refined ribosome. Lso2 is highlighted in red. The class with the best resolved SSU-head, Class 2, contained additional density for an E-site tRNA (sky blue), and was refined to an overall resolution of 2.93 Å. Multibody refinement yielded maps with resolutions of 3.28 Å for the SSU-head (EMD-11437-additional map 1), 3.04 Å for the SSU-body (EMD-11437-additional map 2), and 2.83 Å for the LSU (EMD-11437-additional map 3). These maps were combined using PHENIX combine-focused-maps (EMD-11437). *The inset depicts a superposition of Class 1 and 2 to visualize the 2 conformational states of the SSU-head. øTo estimate the percentage of ribosomes bound to Lso2, a mask enclosing this region was used for a 3D classification without image alignment. Class 1 shows clear density for Lso2, suggesting that 91.8% of all ribosomes are bound by this factor. (TIF) [file pbio.3000958.s001.tif]

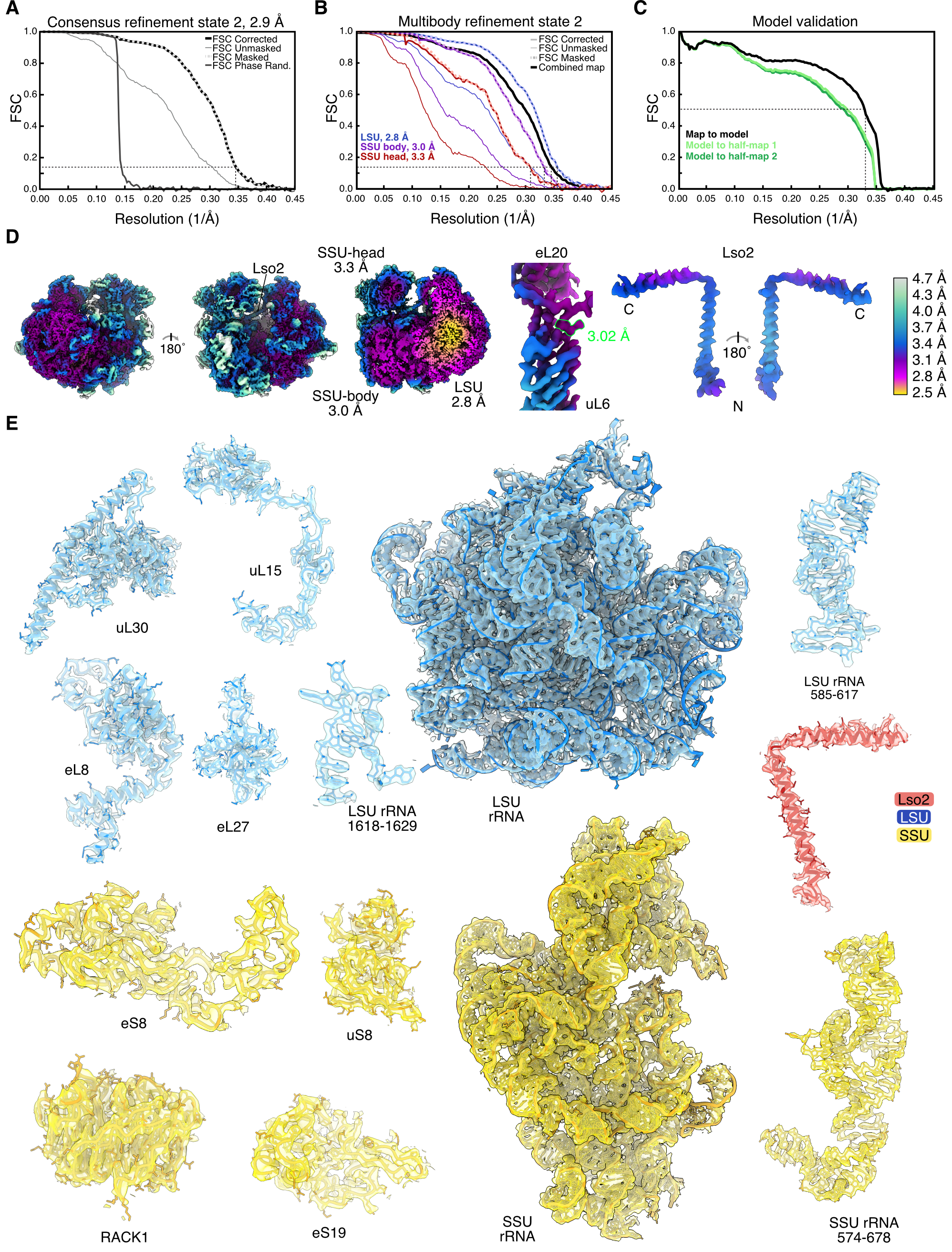

Supplement: S2 Fig — (A–C) Fourier shell correlation (FSC) curves of the consensus refined state 2 (A), the multibody refined maps and the combined final volume (B), and map-to-model cross-validation (C). The thin dashed line indicates an FSC value at 0.143 or 0.5. Curves were obtained from RELION-3.1 [41] (A and B) or EMAN2 [53] (C). (D) The final focused refined map (EMD-11437) is shown (left) next to a core-region cross-section (middle). Densities for eL20, uL6, and the bound nucleotide (highlighted in lime) and Lso2 (right) are displayed in isolation. All maps are colored according to local resolution. Local resolution was estimated using RELION-3.1 and visualized in UCSF ChimeraX [52]. (E) Selected representative cryo-EM densities superimposed with the corresponding models (PDB 6ZU5), colored in blue (LSU), yellow (SSU), or red (Lso2). (TIF) [file pbio.3000958.s002.tif]

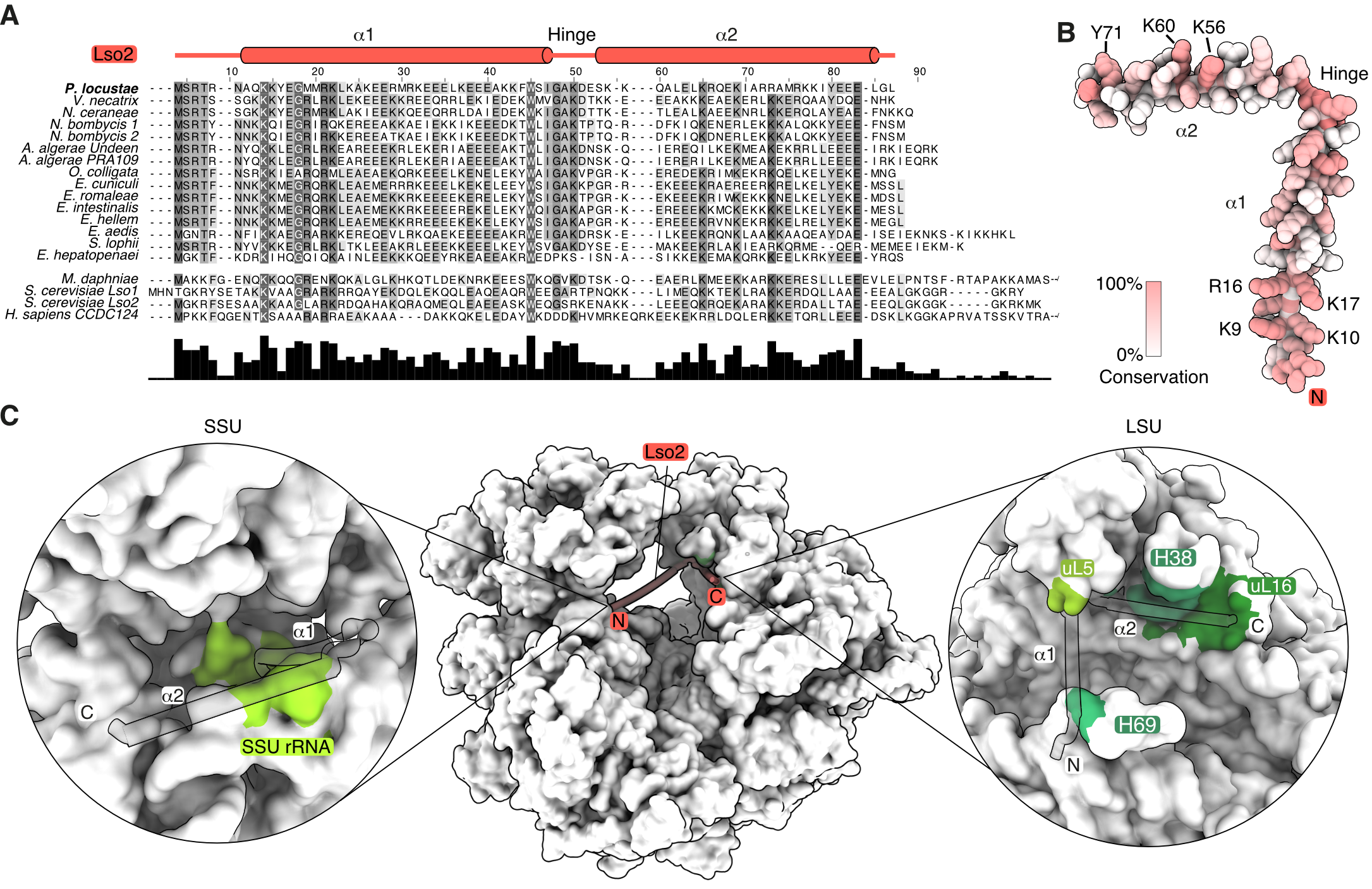

Supplement: S3 Fig — (A) A multiple sequence alignment of Lso2 from microsporidia and selected eukaryotes. The related S. cerevisae Lso1 is also included. The C-terminal ends of M. daphniae and Homo sapiens have been truncated. The domain architecture of Lso2 is presented on the top. (B) Lso2 shown in isolation with side-chains as spheres, colored according to conservation from white (variable) to red (conserved). The conservation was calculated with Homolmapper [54], using the microsporidian sequences shown in (A). (C) Lso2–ribosome interaction interfaces (shades of green) were obtained using EBI PISA [55]. (B and C) Molecular models are shown from PDB 6ZU5. (TIF) [file pbio.3000958.s003.tif]

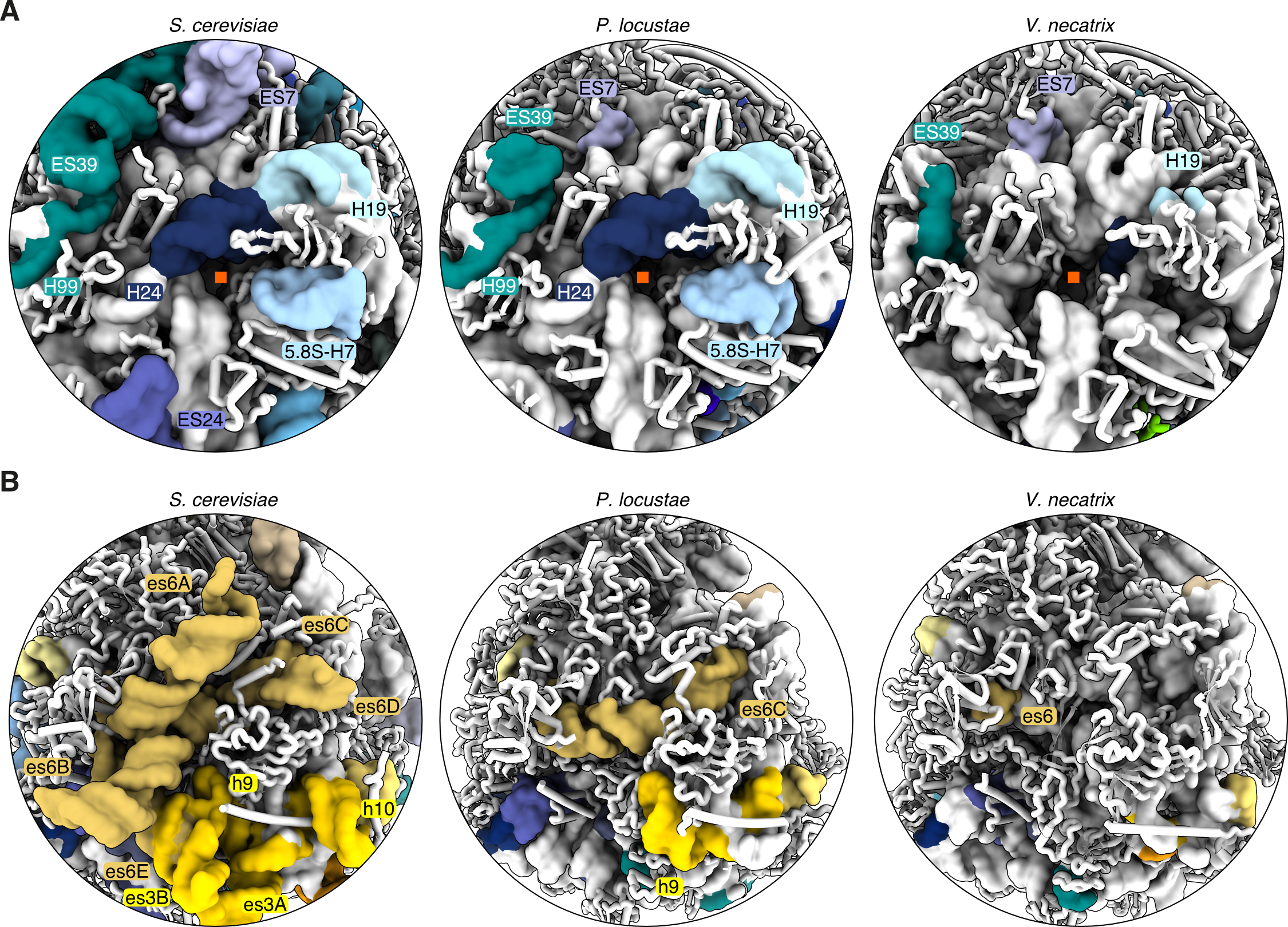

Supplement: S4 Fig — (A) LSU region around the polypeptide exit tunnel, shown for S. cerevisiae (PDB 4V88 [19]), P. locustae (PDB 6ZU5, solved here), and V. necatrix (PDB 6RM3 [9]). Eukaryotic ESs and rRNA helices diminish from left to right. Peptide exit tunnels are denoted by a red square. (B) Reduction of the SSU ESs es6 and es3. P. locustae again represents an intermediate state in this reduction. (TIF) [file pbio.3000958.s004.tif]
