# Supplementary material for: Differences in structure and hibernation mechanism highlight diversification of the microsporidian ribosome
Source: PLoS Biol. 2020 Oct 30;18(10):e3000958. doi: 10.1371/journal.pbio.3000958 (PMC7644102; doi:10.1371/journal.pbio.3000958)
Supplement: S1 Table — (PDF) [file pbio.3000958.s005.pdf]

	**Combined focused maps**<br><br>**EMD-11437**<br><br>**PDB-6ZU5**	**LSU**<br><br>**EMD-11437-additional map 1**	**SSU-body**<br><br>**EMD-11437-additional map 2**	**SSU-head**<br><br>**EMD-11437-additional map 3**
**Data collection and processing**				
Voltage (kV)	300	300	300	300
Pixel Size (Å)	1.041	1.041	1.041	1.041
Electron exposure (e-/Å2)	28.6	28.6	28.6	28.6
Defocus range (um)	0.7-2um	0.7-2um	0.7-2um	0.7-2um
Frames	40	40	40	40
Symmetry imposed	C1	C1	C1	C1
Initial particle images	320,669	320,669	320,669	320,669
Final particle images	124,947	124,947	124,947	124,947
Resolution (Å)	2.9	2.83	3.04	3.28
FSC threshold	0.143	0.143	0.143	0.143
Map sharpening B-Factor (Å2)	Combined map	-25.0655	-21.2595	-21.3032
**Refinement**				
Initial model used	4V88			
Model composition				
Non hydrogen Atoms	165,177			
Protein residues	10,308			
RNA bases	3,910			
Ligands	187			
R.m.s deviations				
Bond length (Å)	0.010			
Angles (°)	1.02			
Validation				
MolProbity score	1.47			
Clashscore	5.17			
Poor rotamers (%)	0.65			
Good sugar puckers (%)	99.11			
Ramachandran				
Favored (%)	96.81			
Allowed (%)	3.11			
Outliers (%)	0.08			
