# Supplementary material for: Differences in structure and hibernation mechanism highlight diversification of the microsporidian ribosome
Source: PLoS Biol. 2020 Oct 30;18(10):e3000958. doi: 10.1371/journal.pbio.3000958 (PMC7644102; doi:10.1371/journal.pbio.3000958)
Supplement: S2 Table — Bolded and underlined sequences were modeled with side-chains while green regions were trimmed but still contain side-chain information. Sections indicated in yellow were modeled with poly-alanine structural elements, and the ubiquitin moiety of eL40 is indicated in blue. (PDF) [file pbio.3000958.s006.pdf]

Chain	Subunit	Sequence
S60	18S rRNA	C**ACCAGGUUGAUUCUGCCUGACGUGUGCGCUAGUCUUGGGGACUUAGCCAUGCAAGCCAGCGAAGC**GGC**GCGGAGCGGCGGACGGCUCAGUAGGACGGCGAUAAUCCACCCGCAGCAGAGACAAGGAUAACCGCGGCAAGCUGCGGCUAAGACAUGGACAGGCUGCGGCGCGGGGAGGCGCGGGCAGCGGACAGGGAGCGGCUGCGGCGGAGUUGCUGGCCCAUCAGCUGGUAGGUAGGGUAAGGGCCUACCUAGGCGACGACGGGUGACGGGGGGUGAGAGCCCGGAACCGGAGAGGGAGCCUGAGAAACAGCUCCCACGUCCAAGGACGGCAGCAGGCGCGGAACUUGCCCAAUCCCGGCGGGGGAGGCAGCCACAAGACGU**GGGGGCCCGGAA**CGCACGGCAAAGGAGUG**CGGGGCG**ACUGGAGGGCAAGUCUGGUGCCAGCAGCCGCG**GUA**AUUCCAGCUCCAGGAGCGCACAUGAGUGUUGCUGCAGUUAAAACGUCCGUAGUCGGCCCGGGCGCGCUGUGAGGAAACCGCGGGGCCCAAGGCGGCGGGCAGCACGGGAGCGGGGGCGACAGGAACGGACAACGGCCGCGGGAUCCGGGGGCGAGAGGUGAAAAUCGGGGACCCCGCGGGGACGGGCGGAAGCGAAGGCGGCGGCCGGGGACGUGUCCGUUGAUCAAGGACGAAGGCCGGAGGAUCAAAGAUGAUUAGAUACCGUCGUAGUUCCGGCAGUAAACGAUGCCGACGGGGCGAGGCCAGGGGGCUGUGUCCGGGAGAAAUUGGAGUGUUUGGGCUCUGGGGAUAGUACGGUCGCAAGACAGAAACUUAAAGAAAUUGACGGAAGGACACCACAAGGAGUGGAGUGUGCGGCUUAAUUUGACUCAACGCGGGGCAGCUUACCAGGGCCGGAUGC**GCGGGAGAUUGGCGGCGGAGCGCCCCCGAGAUUGC**GCAGGGAGUGGUGCAUGGCCGUUUGCAACACGUGGGGUGACCUGUCUGGUUGAUUCCGACAACGCGUGAGGCCUGGGGUGCAGCAGGGGCUGUGCGACGGGCGGCGGCAAGCUGCAGGAGGGCAGGCGAAAACAGGUCAGUGAUGCCCUCAGAUGCUCUGGGCUGCACGCGCACUACAAUGGCGCGGCGAGCGGGAGUGCCGGGAGGCGCGGGCGAGGCCGUGGCGCAGCAGGGAUGGGGGCCUGGAAGGGUGCCCUGAACGAGGAAUUCCUAGUAGCCGCGGGUCACCAAGCCGCGGCGAAUGAGUCCCUGUUCUUUGUACACACCGCCCGUCACUACCUAAGAUGGAUGUGCAGGCGAGGAGGCUGGGCAGGCACCCGAGCCUGUGCAACUAGAUAAGGUAUAAGUCGUAACAAGGCUGCAGUAGGUGAACCUGCGGCAGGAUCACUA**
L50	25S rRNA	**ACACACCCCGGUGGGGGAUCCCUCGGCCUGCGCGCCGGGCAAGGACGCGGACGCACGCGAUAGACGGCACGAUCCUCAGACACGACUGCCGGUCUCCGACAGCGGCGCAGCCGCAGACAACCCCCCGGACUUAAGCAUAUCACUAGGGGGCGGAGAAGAAACCAACAGGGAUUCCUGCAGUAGCGGCGAGCGAACAGGGACGAGCCCGCAUGGCAAUCGGCAUCGCCGAGUUGUGACAGCGCACCGCGAACGCCCCGGACAGGGCGGCCACAGAGGGCGACAGCCCCGUAGCAGCGCGCAGCGGAGCGAGUAGCGCUGCUUGGUCAUGCAGCGCGAAGCGGUGGUGGCGCCAUCGAAGGCUAAAUACGCCGCAGGACCGAUAGCGCACAAGUACCGCGAGGGGACGGCGACGAGCAGCCCGCAGGGGCGGCGAAAGCGUGAAACCACCGGGGCGCCCACUUGUGGGCCCCGUCUUGAAACACGGACCAAGGAGUGCAUGUGCGCAGCGAGUCCGCUCCGCGGCGCAGCGAAGGCCAUCGAGCUGCGCACAUGCGACCCGAUAGGCAGUGAACUACGCCUGGGCAGGGCGAAGCCCGCGGAAACGCAGGUGGAGGCCCCGAGCCGUUCUGACGUGCAAUUCGAUGGCGCGACCUGGGCGUAGCGGCGAAAGACCAAUCGAACUGCCUGGUAGCUGGUUCCCUCCGAAAUGUCCCGCAGGACAGCGGGCGCCCCGCAGGUCUGCCGCGUAGAGCAAUGGCGCGGCGUCCGG**CAG**CGCCGGCGCACCCCCAAACUGCGAAGCGGCAGGGCGCGCGCAGCAGCGUGCGCGCGCACAACUGCGGGCGCCUAGUGGGCCGCCGCUGGUAAGCAGCGCCGGCAAUGAGGACACAACCUCGUGCGCGGGCAAGGGACCCCAGCUGCGCACACAGACGAAGGGCGCGGGCGC**GUCGCGACAGCAGGGCGGUGGCCAUAGAGGUCGGCACCCGCUAAGAACCGUGUUGCAACGUACCUGCCGAACAC**GCCCGCCCCGAAAAUGGACGGUGCUCAGCGCAGCCCCGACCCCGCGCACGCACAGCGUGGUAGGAGGGCGCGCCGGCGCCGCAGAAGCGCAUGCGUGCGCAUGCGUGGAGGCACCCGCGGCGCAGAUCUUGGUGGCAGUAGCACACUCGGGCGCGAGCCCCGAGGGCCGGGAGACGGGUUCUUCCGCCAGGCCGCUCCGCGGAAGGUGAGCCGGGUCCUAAGGACGCGCUGGCCCGCAACCGACAGGCAAGCGGGCACACAUUCCCGCGCCGUGUGCCAUGCGGCAACGCACCGUGCGCGGCCGGGCGCAGGGCUGGCGCCGGGGGCCCUCCUCCCCCG**CAAA**GCGGCCCGCCUGCGGACUCUUGCAGCACGAGGCAGCCCGCGCCGC**GUGGCGGGGCCGUCGCCGCG**CGCCAGGACUCGCCCCCCGUGAAGCCCCGCGCACGCACACACACGCCCGUACCAAUCCGCACCAGGGCUCCAGGGCGCGCACCCCACGGCCAGGGCCCACGCAGGUUUGGGAAUUCGGCAAGCUGGAUCCGCAACCUCGGGACAAGGAUUGGCUCCGGGCGCCGGAGCUGUCGCUUCCAAGGGGAAUCCGACUGUUUAGUAAAAACAUAGCCUUGCGCCGCACGCAAGGUGAAUUCUGCCCAGUGCCCGGGACGUCACGCCGGCGCGACCCGCGCACGCACGGGUCAACGGCGGGAGUAACUAUGACUCUCUUAAGGUAGCCAAACGCCUCGUCAUCUAAUUAGUGACGCGCAUGAAUGGAGCAACGAGAUUCCCACUGUCCCUACCUGCUCCCCAGCGAACCCACUGCCAAGGGAACGGGCUUGGCGCAGUCAGCGGGGAAAGAAGACCCUGUUGAGCUUGACUCUAGUGUGGGGCCGCG**GCGCGCCGCGCCGGCGUAGGCAGGUGGGAGGUGCGCCGUGAGUGAAAGACCACUGCGCGCGCGCG**CGCCCGCUUCGCGCAGCAACGCCCCCAGAUGGGGAGUUUGGCUGGGGCGGCACGUCUGCUAGACCCCAACGCAGACGUCCUACGGUGGGCUCAGCGCGGACAGAACCCGCGCGUCGAGCACAAGGGCAAACGCCCGCCUCACGGCGCCCCCCCGGGUGCCGGCGGGAAACCGGGGCCUAGCGAUCCCUCGCGCAUGCACGCCGCGUCGCGGGGGUGGCUGAAAAGUUACCACAGGGAUAACUGGCUUGUGGCGGCCAAGCGUCCGCAGCGACGCCGCUUUUUGAUUCUUCGAUGUCGGCUCUUCCUAGCAUGGCGUGGCAGCGCGCGCCAAGUGUUGGAUUGUUCACCCACUGACAGGGAACGUGAGCUGGGUUUAGACCGUCGUGAGACAGGUUAGUUUUACCCUACUGAGC**GC**GGACACACCGGGCAGCGCGGGCUAGUACGAGAGGAACGCCCGUGCGGGGCCGCUGGUCCGCGCCUGUCCGACAGGGCAGGUGCGCCGCUACGCCCCGUGCGUGUACGGCUGGAC**GC**CUCUAAGCCGGAGCCGCCCCCCCGUGUGUCUAAAC**CCCUG**GUUUCCGCCCCCCGCGACCACGACGCGGCCGGGGGCUGGUGCUGUGCGCGUGCGAGCUCUGCGAGCCGCUGAGGCUUCCAGACCCCUGCGGGGUGUU**
L70	5S rRNA	**AGCUGCGGUCAUAUCCACAGUAGAGCACCAGAACCCGUCAGAUCUCUGAAGUUAAGCCUGUGAGAGCCUGUGGAGUACUUGGGUGGGCGACCACCUGGGAAAUGCAGGUACUGUAGCUU**
LNN	Lso2	M**SRTRNAQKKYEGMMRKLKAKEERMRKEEELKEEEAKKFWSIGAKDESKKQALELKRQEKIARRAMRKKIYEEELG**L
SA0	uS2	MQKEIEIPEDFVKAIILSNAHLGSTTATTNFKQYIYGRRKNDLIHVIDINEMWKKLIIAARLFVSIEDPSSIIVASNKKFGKRAVLRFCSETGATPLIGRFIPGGFTNQVINDVKESRLVVVSDTYADKQTVMEASYVNTPCIAFCNSDNSLSRVDVVIPMNNRSPLSIGCGFYLFAKVIKYIKGEESFDVDVRSEIELYFYRDPVELDMIIQEQKEKNETSFQVKDAAPEVPAEAEQWADA
SB0	eS1	MAKKDQPVTKKSLKKGLKKKATD**SFAKKEWFSLRIPSGFVTAEPGKTLCTKQNTKANLEKYLLGRNFEVNQGDLNPGNEESAVRKFRFIVNNVNGRIAESEFNGMELTTEKKRGIVRKWHTLIEAFRDVCTKDGYHLRVFVTALTKHR**PGSVS**RTCYAKTSDVKKVRRLMFEVIENEVSGSELAKVVKKLSVEKLGKEIEKKASVILPLQNCYTAKVKVVKRPR**PEVIRN
SC0	uS5	MESSVAGRNTGPLPRKKEEAVRPRVPRQQDK**EWVPTTDLGILVKMGKINMEDIFQYSLKIKESGIVDHLLKGQLKEEVIKIKSVQKQTRAGQRTRMKVVVVVGNGAGYVGIGTKTSKEAATAIKGAIERAKCAIRPVRLGFWGNNYGKEHTVPCKATGKSGSVIMRVIPAPKGSGIRAGSVPKKIFQLAGVKDVFTLSKGQTSTTENFAKATVSALDKSSCFFVPEFWREKEPELNPLSRYSRLIKEYREKI**ARK
SD0	uS3	M**VSTKQVLSKFIEKGIMKAELNEFFSKEFEDEVYSGMEMKPHEIPVQITVRVNKTHEVIGERKSRLNRIKHVLMERFPLLRTGVEINVEMVKNKGLCPKTQAEYIKAKLIENVPFRRAVNSVMRSIKESGAQGCVIIVSGKLKGQRARSVKYADGLLIHTGSSKEDYVKEALSVVHLKQGVIGIKVRIMLPYDPENVNGPSKVISDKIKIFEPK**ELY
SE0	eS4	M**TRGPRRHLKRLNAPSSWQLDKLGGKYAPKPSSGPHRLSECIPLCILLTRKLEYATTTKELKYILRKKMIKVNGVVRTDKDFPVGFMDVISIDTTNENMRLLYSNNRAFMLHNISSEEATYRLCRVSKKKMGIGGIPYIYTQDGSTFRYCDPKINVNDVVKVNIKEKKVVGFLPFKSGMKVFITKGKNLGCIGVISGIEKHMGSHDIAYVVDAKGRSFSTRTSNAFIIGDSESYWISLPEGEGIKISELEKSNIKYGE**IASVPVAEE
SF0	uS7	PMSIACDSGE**LLLFDKYSRDEVVVNDRSLKNYIFLRSLSSYPHTASRKGSRQFGKGEIPIEERFVCGLMRRGRNSGKKRLAMAALRNAFIIINTLTGKNPLQVLVDALVNCGPREDCARIGKGGAMKRTSVDVSPLRRLNVAIFLVCKGIRQACMKSTKTIAEAIADELIAASRNSPNSFGVKKKDEIERMAKS**NR
SG0	eS6	**MKLNVAYPAKGTQKMIPIDFQTSMRLYGLRLGAQVEGELIGEDFRGYVFKITGGDDVSGLPMKDGVFTDKRVRLLLKKGNKGYRCREKGVRKRKSVRGCIISGEIAVVSMIILKEGEKP**IEGLTD**RVVECSHLPKRASKLRKLFNIPKDANIVEFIKEQIRNNSDSEKKIRYPRIKVTRLITPMRIERKRKRLEEKEARKIKSENERKEYMKKYYPEKI**ESL
SH0	eS7	MSEQTEHEKKVAQEIKKLYSQDMSGEFGDKLSDIRIQTIDREQGKKILLIRVPEEVLEVVHIGYSKLSGKLKALYPDFHTLVIRNTPIVNVSSIKGNVYRI**DSKNTKKSVHENWIRDLCYPALVEMRKTDVFNGCERIESALVSSNSNYSDDDFAAMECAFKTLTGRTIHYGNIFY**
SI0	eS8	M**GLSRSNRHKRTKTGAKKPIFCKKRKFHMARQPSNTKIGEDKLQELRCRGGVIKKRALRLSSGNFTCRTHGFSYKTKIMQVMYHPSSNELMRTNTLTKSAVIKLDSKQFKEKFDSILSTDSELKNTDPLFFSNLDAGKLYGVIVSRPGQCGRADGYILQGDELNFYINKFK**KKAR
SJ0	uS4	M**ARPKYSKRSRVPRKPYEKDRLIDEMKLLGEFGLKNKRELWTQEKICDDIKKRARDLLISTDESEIIINGRALLNKLIKLGIFSDVDFKDMDDIKRNLEKVLDLTASHFLRRRLQHRVFEACLAKSVHHARNLINHRHITVRGCVIDKPGYMVFAENEGHIEINK**FSSVGGAKPGRSKKKHAVAQGE
SK0	eS10	M**LIPTKNILAVKRHMLEKRCIVVEDTPKGNHPVLNIPNLQVLNIMKSLVSKRYVKKTFCWRHGYYFLE**DTAVDVLKDQLCMSDDEAAGLYAPSAVVSNIKAQ
SL0	uS17	MGDILDKA**KTYPKQKKPHL**VSRREGKDI**APERYYKNIGMGYNVPETAINGTYIDKKCPFTGNVTIRGRIFKGEVFKMKQEKTIVVVKRYFHYNKKYKRYERRNTKFNVHLSPCFFGLVRVGDTVTCGETRALSKTKHFAVIDYKQKDS**EDGQYAQFTGFS
SN0	uS15	M**ARMHSKGRGVSESTVPYNRVRPHWVTQSKDEVVGTILSLSRKGLKPSEIGKIMRDEYAIGHVRNVTGMNILQILRAHGLAPAIPEDLCALLDKRAGILSHLNTFRNDKSARYRLNLVESRMYRLARYYKRTMVLPATWKPHA**ASK
SO0	uS11	MTEDIS**TKDLVFGVAHILATSNDTFVHITDLTGSETVCKVSGGMKVKAHRDESSPYAAMLAAQDAAQKAIERGITAVHVKLRGRGGVKSAIPGPGAQAALRALIRNGLKLGRVEDATPTPTDRTRRKGGRRGRRL**
SP0	uS19	MEGDVEKERRQFK**KYTYRGVPLSELVKMPLLKFSELLTSSKRRRLRRGISERELDLLKKCALSKKNAEGTQEKPDMVKTQARTMVIVPQMVGCVIGVHNGRVYVPVEIKPEMIGYYLKDFSDTKT**CSTHGKPGVGATSGSKFVPLK
SW0	uS8	**MNRLAATCKAINNANRSGKRQVLIRFVTKTTRKFLEKMQVCGYISTLTYIDDHRQGKVIIGLNGRLNKCGAICPNYCLTLKDVEKFKDKILPARQFGHLILNTSKGMIDHNECVTMKSGGEVLGFFY**
SQ0	uS9	AIPM**AKKVIAIGQKKTAKARSECMEGDVFSVTVNNVPLRLLPDDMMASKLRDIITRVGLKYFENLVFSITVRGGGPVSKIYAVRQAFAKSLVAYYGTYMDEWTRQEIRSNLLSFDKFTLVADPRRAEPKKY**GGRGARARRQKSYR
SR0	eS17	MG**RVRNSNVKRAAKNIVEKYFPKLDCDFHHNKRVLESVTEIQTKRFKNQIAGYVTHLYTRVEKGKVKKIFI**KKHEEERERRENMIPSSSIMDTEKIEVDDITMQMIKDYCYRGNYVVSVSEEQ
SS0	uS13	MEGRKS**LFETFTSEPAQIQHIIRMLNTNIDGSRRVVHALTQIKGIGKRMSDAIVKRTGVDASKRMGALSASELELLQDGIQNPAKYGIPRWMFNHQFDAVDGTDSHLIGNQIDANLRFYIEKGKKMKHIRVCRLARGLKVRGQRTKSNG**RGGKTIGVSRKK
ST0	eS19	**MKELFMVRADALNEALAGYLKSKNIVKLPENFDIIKTSHGKQRAPSDKDWYYKRAASIFRKLLIAMHEEKRIGVKRLSRKYGCAKNRGSRPSKHVDGSRGHIRKIMQDLERAGLIVKDGNYRKVSDMGIIETGQLISQI**KE
SU0	uS10	MELEIDEKKY**SVDINLEGTSVEEIEKVCRDFYAYAKTLVPDMGGVKRLQTKKGKITTRRSPCGEGTNTWSRYRISVHRRSFSAHLYPSVLEKFATFLKGSNVEISLVIK**SN
SV0	eS21	MS**FEQRICTFTGRPIPHTDNASVQLTFV**ELDSDGRATDKV**LIYNVCGKIRKDGTIDEILTNTLAQ**N

SX0	uS12	MKGLYCAKKLKRNRQAKRRNDPTYRKRLLGTKYKQDIIGVAPQAKGIVLEKIEVEAKQPNSAIRKAVRVQLIKNGKKVSAFVPYDGAINDIDMNDIVTLEGFGKKGRSKGDIPGIRYKVCKVQNVSLLALFRGKKDKPSR*
SY0	eS24	MSLETQICMMRENRPLQRREVVLSISHPKSRTPSKEDICSQISSLFKVRKELIIVSGCSTRFGTHQTKCKVRIYESPEMLREIERDFVVKRKTGEEVRKKKARRVRKKERKEKAKIFGTLRRHLKKAEKRAQK
SZ0	eS25	MVKKVLESKEKKAAKIASTSNKEKKKWTQGKTREAVRRSVTVEADVFSKIERDVAKASLVTAPSVAEKFNLNVGVAQKILEHLCAGGVLCCLSRNSRLRLYSRAQKVGARPADTTLPAAEAPAQTE
SAA	eS26	MPVKRKNHGRAKKNRGHVKLIRCDNCASAVPKDKAIKRFQIKSLIEAAAHDDVRTATIYEEYEVPKFFHKNQYCVSCAVHLKAVRCRSAEGRKDRSNPHARVKQ
SBB	eS27	MAIKDLAFPTAEELRTTCKRKRLIPQNNSYFLYLRCKSCDIIILAYSHSQTRRTCPGCNMVMLMPKGGKARIEGDVKIKKIKRLVE
SCC	eS28	MTDQVFLGEVMQLYNKTGPGGSITLCQLKLEDTGRMLHRAVIGPIKIGDVVTLLDCEREHRRGRF
SDD	uS14	MEENIKIKPGSYAGRVDTRKFGRGSRSCRACFTHRGIIRQYNLYLCRRCFREYALEIGFKKVD
SEE	eS30	LESKGSIARAGKVRNQTPKVEKQEKPKALTGRARKRALYERRLEANFFETRKMKMNPNFS
SGG	RACK1	MTTTARLNEVATFSGHKDAVMALGTCETPDAKLLFSASRDRRVLGWSLGEGAMFGRIVKEFTKHGHSVNDVAVARSGSFVVSAGCDGLGRIIDVQSGERTLLRGHESDLTCAAINCQENKIVTGSVDKTLRLWNMCGELQHTFDAAIECAHEDWVMCAEFRPLDENEVVSGSVDGTVKVWDIDARVVKSTFFDGCLIQSCAEGSEYRTRPVADGSFAVRALALSADGSCCAYGGSNCKTYILNLAESEAIAAFETDTPVSALAFGLTDVILACGTQDKIYIWDVVSNCLLAVADLSAHGKRVRCRSLVWTTSNLIAGLGNGKILVFEFVR
LA0	uL2	MSKVIRRLRLLKPHHRPKAMWKVGETRIPVVSETMHGVVSEIVHERGKVAPLAKIRVDTGKCVRRELLVAVEGNYVGQKVEIGDSVPVAVGNALKLKNIPEGTGVCSVERRPYDGGKMAKSSGAYVTVVGHNRDTNITTVRLPSGEKRSVSSECRAVVGVIAGGGVNEKPLLKASRAHYRAKARGLYWPTVRGVAMNPVDHPHGGGNKQHIGHPSTISKHAPPGQKVGLVAARRTGLRRGSKKVLNK
LB0	uL3	MSCRKFEAPRHGSLAYMPRRRARSVKQSIRAFEKDNPEDPIHLTAFYVYKAGMTHVVRNKAMNDKKGTIKEVTESVTILEAPPMVVFGIVGYVNTPQGLKINKTLLSSHINESVLRRFYRKFYLSKKRMFSSARRKAGQKELDADILVLKDSDVIRVLAHTQVEKIKSIRTKKAHISEIQVNGGTVNDKVEWAVSMLEREVKISDVFSTNEFVDTIGVTKGKGFQGVTKRFGTRILPRKTNKGRRKVACIGAWHPANVLRTVPRAGQLGFHRRTELNKLIYLIGNGKEEIKTDFDPTLKSINPMGGFPHYGLVNNDFLMVKGGITGPVKRVLAIRKNLIGKKNNENIQIKFIDTSSKIGSGRFQTSEEKRAFFGITKKDVSEEIK
LC0	uL4	MRDTVNCYGIDGETVEKQLEMPDVLRVPIRKDLVEDAFRCVRMDNRQPYAVSPNAGMQHSAHSWGTGRAMARVPRVSGSGTTRSGQGAFANFCRKGRLAHPTKVIRRWQRKFNLNAKRHAEAMALAATAIPPLVESRGHRIAGVKMIPLVVSNSIKEIKSTKEAFEMLKRFGLAEELARVKESKCIRAGKGKMRNRRYVMKKGLLIIYDNQSDIQKAFRNIAGVDLACVDSLSLLDLCPGSHLGRLVMWTLGAFEKLNEIYGQYGKEAPLTSGYFLPTNVVSKDDVESLFFSDEIQAFLDVPNLIKYEKTSRKPETIESLNPYLNLMESN
LD0	uL18	MTDNKIKKSYFSRFQTKLRRRREGKTDYKHRYNLIRQDVNKHGLMKIRLVVRITNSRIICEILRAHVDGDRSIAYADSTELKRYGITFGLKNYTAAYATGLLVACRYNNKIAGEGPRPECYLDIGLRRSTRGARVFGAMKGALDGGLVMPHSLKRVPGYVSEEEFDSEVFRNKLFGKILAGYMKEMMENYPEKYKKTFQEYIKKGINPDDLENIYENAFKKIREDPSRVSKTHGDYSIFKEFKRVRLSKEERAARSRAKLLDIEVK
LE0	eL6	MIDTSKLSMLSRRGIKVIPEEAGLYPSDDLPAYIEKLEKRMQKKPRVRRTDLVKGCIVVVLEGDFTARRVVFLKQVENNKALCCGPAPINNVPFFVIDERYLLRTSTVLDLKEDVNIDISTVFESKRGVVADRMDIDANSDQKRIENAIVDAVSSIRFMKRYLATPFKMPKFKSVSSLKF
LF0	uL30	MDQEVPYSYRRKMEYEERMEVLSRKMNEEYQARREENARYALQRTKELIAEYKEHMKKEEDARREAEARGAFYVPKEPEFYVVVRIRGIHKVPPRERKILELIRLKKPNHAVFVRNNAPMRAMLHKVRAYIAFGFADIHLLRTLVYKRGMAKVSKRPLANNSSVFKIGQDMRLNLTNEAIEDHFGGRIRCVEELIYQIYMGTDLFKKANNFLWPFTLCSPRKGFGGRKAKDITQGGSTGCHYDKIGNLIYRMIE
LG0	eL8	MASQAKREMIERRRNPRDLDQKHQREERKALIAERVHLYRNALRIPPAVYQFSTFLNQQDMDQVLSLFRNYIPETRAEKKKRLMSENPRAGRKPILIKFGLKHVTDLIERKEARIVIIASDVDPIEVVVFLPTLCRKMGIPYAIVNGKKELGTLVHLKSTSCICLCDVAPKDSVEFKNILAKINAIFMDNYEITMKKWGGGALLRDKPQESSAPQA
LH0	uL6	MKRLLCEEKVEIPEGCSVEILERVMTVRGKRATAVRDLSHFVLTMDVHEGHVRLRLWNGTNRERSKLITCASVIRNCIVGCMSGYEYTLKVVYKHFPMSVAIEDDGKTVVVKNFLGQKHARRYKMRGDSIARLGTEKDTFVVEGSSLEDVSQSAGTIQENCQVKKIDSRTFLDGIYFLSRNVVGAK
LI0	uL16	MGRRPARCYRYCKNKPYPKSRFCRGVPDPKLAIYDLGRKRARVTEFPLSVHLVSNEREQLSAEALEAARIAANRYMIKHAGKDNFHMRIRVHPLHVIRINKMLSCAGADRLQTGMRGSFGKPYGRVARVDFGQEILSIRTKDAFKAVACEALRRAKAKFPGHQQIKVSCAFGFTGISREEFERLRKEGKLISQGSHVTIIKEKGSIVGYKEKLSKAIQ
LJ0	uL5	MTEQLENPMRQIRIEKLCINCCVGESGEKLNRALKVLEQISGQKPCTGKARLTIRGFGIRRNEKISAYVTVRGEKAREILNNALKVKEYEIKKSSFSNTGGFGFGIDEHIDLGIKYDPSIGIYGMDFIVVLSRPGLRVSKRRIKKSRVGNKQRISKEEAMQWFVKNYDGVLLNK
LL0	eL13	MKHNNTLPNNHFKKTAIRFKTWFNQPAKKEKRIQLRKEKAEKMYPMPVEKLRPIVRCSSIRYNIKQRLGRGFTPEECRAAGLDYNYARTIGIAVDMRRKNMNKETFDQNVERIKVYTSRLTFYKDRKEAKASGAAQHIGKIMPPKKTPPVVQTIKVEEIAQKFPTK
LO0	uL13	MINKEIIVDGTGHIAGKLGTYIAKKLLEGYTITVLCAESIVLTGPIHRTKLRYKDYLNKRCLVNPLRGPFHYKEPSKLFMRLVKRMVPYKKKRGAAALQRLQVFEGIPEKFENTERSICPRALLEYCANPIRKSATYGKLLSEFGWKHLNITEEMKKKVLQREEKAKKEKDARMEEIQRIRESSSFNKEVEEIMSRIE
LM0	eL14	MAKNFVQVGRLATPRFLAQRNILYVITDIQDDSMVIVQDAQGTRKLVSVKSLHLMDDVVEIHRDMSVEEVSKKIPQQKEEEVSENDFERFKRELRTEIEEEVLRSRGF
LN0	eL15	MSASEYLREIRKKKQSDLARYLGTIRNYEFRLNTAVHRAERPTFPERAHKLGYKAKQGICIFRVRIRRGGRKRLVNNGNTRGKPVNAGIYQLKPANSLQSMAELKAGKRAGNLRVLNSYWVGQDGVYKYFEVIMVDPNHNAIRNDPKLNWICKSTMKHRECRGLTSASRKSRGLGKGIRYNHTIGGSRKAAWRRRNTVSLRRYR
LP0	uL22	MKKKYAPEIHDPGNTVKCRIDNARVSFKNTRETSRVLVNRKLGDALNYLYDVIKKKQCVPMKRYARGVGRTAQAKAFKTQRGRWPVKSAKFFIELINNLKVNAVNKNLNPDEMVIKNIIVNKAPIIPGRIHRAYGRINPYNSHPCHIQMIAVKKMVAVPKAIDGEYAAEVV
LQ0	eL18	MVATAFASKKYVSRGRKKLVSRNIYLEGLAALYERIAKSSANEIVHKIAAQLKMSRNNRPPMKVSKLANISEKYPGKVYVVVAKVLDDNCFLEVPKMQIVALQFSKSAKEKIEKAGGSTHTLDQLFEVAPGLENVELFRGDLTARKAYKYFGAPGDRHSRTYPKTTSKGKNREKRLLK
LS0	eL20	MWRCGGIKEYRIYGSKMPTEQEVAPQIFFHNVFAKNEIVARSRFNNLMKTKYKIKSGKIVILKIEELVEDLKDMKIKNYGIQLVYRSKKGIHNMYKEFRSISRCKAVEMLFNDMAGRHKAKRDDIKIVSLKELRSEDLRRDRVIQFTKDDVMYPVFKKVLNSKYDFVLKGTNIFN
LR0	eL19	MTKLPTIRRIAASIFKCGKNKIWFDPGETPRLASSSTRMQVRRLIKDGVILRKPNTVHSRWRANKRAEARKKGRHMGIGKRKGTKNARMPEKRVWIKKIRGQRASLKEMKKSGHITPEEFRQYYMQAKGNMFKSLKVMEDHIEKKREEKMRIKDLAAQAAALRMKK
LT0	eL21	MRSNGYRRGTRHRFSQAFRKHGMPKPSILTRVFKKGQYVDVVVNPAIHKGMPHKFFHGRTGKIFNIDKRSIGILMNKRCGPRYVEKMVIARVEHVRPSRCNEEYIKRRTENDRLRREAAARGEKLGSLKRKPGGPRGAVLISTENNTPIEIRNEPYFEVY
LU0	eL22	MNSVQAENDTRCYELNCSVLVKDSLLSTEDLNSYLEARMKVNGRTGNLAGNIDLVCTEESILVKPRVRLSKKYLKYLVKKFLYKKELIDWVRILSTGKSSYKLAYYRVVSNKYE
LV0	uL14	MPSKHVLEDRRPKIRSTCGVQVGTRIACADNTGAKIMQIIGVKGVRGRLNRLPAASVGDVVLCSVKKGRPDMRKKIVLCVVIRQKKAWRRRDGSHICFEDNAAVVITNKGDPKGTQIAGPVPREVADIWPKISSNAPAII
LX0	uL23	MEIKRNNPKYTRKAVTQPATGHPADIIRFGACNEKAVRLIENNNTLVFICDKYATKPQIGNAVTRFYKVPVEKVNTARSIKGYKKAYVKLKNEGDALKIANEAGII
LW0	eL24	MVKEGVCVYSGYEVPKGSGLIRVTNDTRSFLFLNKKVQSLSNRKINPRDVAWTAASRAFHKKGMKKVVRKEAEIQVVKEVRGFPSVPKSIIVQPKRDDDQKKKAERGAETFRAQKKVTKAEGRKMKADGRWQR
LY0	uL24	MKFNKEKTASRRKNRKAHFTANSTERRIRMSSPLSKELREKYGFRSFPIRRNDKVVVMKGRFKGNTGTVVEVRRKMYKVYVDSCEASKMNGRKVRVGIDASNLKIIELYQGDGRDKILERKMNNRRMQMERAAKIKMEQK
LZ0	eL27	MLLKPNTVVVALKGRFAGKKGVVVSASEDGRKILVAGIEKMPQPVTDDMSECKKKRLSRMSAFIKNYNSRHLLATRYYGDVGLGAIDFSRIFENFESKKMAIDAVKKAFINANKQKKAAWLFEKLVF
LAA	uL15	MYQNFYPMASKDKKTRKLRGHVSHGHGRVGKHRKHPGGRGKCGGMAHRKTLFMKYHPDHFGKRGMNCTHLKKNARYAPPINVSKLWSLIPKSQLETIMNDNTIAPIINCRSFGYHIVRGGGQLSLKRPIVVMARYFTPKAVSMIESLGGRCIISP
LBB	eL29	MAKRKNHTNHNQNRKNHRNGIKKVKKSAPSFRGLNHKYLRNMLYSRKYNNIGRAAYEAEHGPQQ
LCC	eL30	MSRKRNVTDGLAFKLPLAMRTGVYKVGYKSAIKLLQAGRTKYIVAAANFPSVKRKLLEYYAALSNNVPVVIFKGSNNELAKVCDHHYRIGVISILDDGESGLISAGTQ
LDD	eL31	MASLSNKAVEMTVNLGKLARKASWRYKAPKCIYYLKKFIRSQFKSENDILIAPEVNKYIWRHGIKNIPKRMRIKIERGPSNKNPELNVFRVCLVNVNTFKGLQSQSYTE
LEE	eL32	MEPYLRVPRQRKTKHKFNRFHSDRFKRVKKSWRRPKGIDNRVRRKLSGAIKMPNKGYGTDKLAKHMVHGGFRLVQIRNVGDLLPLISQNRFYCAEVAHSVGAKKRIEIFYKAMEYGIHLTNGKARLVEENKE
LGG	eL34	MVQHLTHRGRRTYNNASNQKKIRRTPGNRRVYIPKKKPGRVPKCVKCRSKLRGIDICRPAAFARLRKSQRTVARTYGGNLCGSCLENRILDAFLSAEEQLLLGKKQTNAADNN
LHH	uL29	MKIRTLELREKSVEELETLAFSLKEELLRLRQQKNTQTLKPHEIRVMRKNIARVLTVRTEKLYAEMYEKHKNDKRMPKDLRPRLTKAKRMALTKRQLNMSVHSVCKRRRAYPRMYFSYSEPVDPKN
LFF	eL33	MKDVFDPCLTAIPATFISHMRGRRIVHPSHSLLRIEGVKTREDAKFFLGNGVVSLYELSGKKMENRGRIRRLHGNSGVVAAKFERNLPPNKIGTEVFVKLYKVEDDDY
LII	eL36	MRKKLRRMRVKYPVTPLVGIEPIPRPGKKHDWNNHGSPMAKAIASEICGLAPYEKKALELIKSDQERKCRRFLKKRLGNLTRTKRKQEALTAIAREQQ
LOO	eL42	NGKWQNNLKVNIPDKRNTHCKKCNKHTEHKVSQYKKSKEGRNGQGTRRYRRKQRGYHGQTKPILRRKAKVTKKLVIKLRCSVCDAKHQQTMKRTKHVEFGGKKKTKGQALTY
LJJ	eL37	SKGTSSFGKKNKRNHILCVRCGSMSYHKQKLRCSSCGYPEKKLRNRGSPKARRRRGEGTGRMRHLKKVRRAARNGFKGNAILRALRNANTTSDSK
LPP	eL43	AKSRGTKKVGIVGKYGVRYGSSLRKRIKAIEISQHAKYECKACGKTSVKREVVGIWKCKACAFTFAGGAFAPTTSAGAHSNSITRQ
LLL	eL39	GSRKTEIIKTRLSRALVRNRPIPVWKRRMRGNTQQYNMKRRHWRRNKLKIY
LMM	eL40	MQVFIKSPGLLEVVSIDRDTTISDLQAMSRYLCNMAFVVNGVLLDKDISLASQGIQDLSTVSAIPLLVGGAGDKQMNENDKALALKRKEAMICRSCYARLAPRAINCRKKKKCGGSNNLRPKKKLKETKKGK-
