# Supplementary material for: Differences in structure and hibernation mechanism highlight diversification of the microsporidian ribosome
Source: PLoS Biol. 2020 Oct 30;18(10):e3000958. doi: 10.1371/journal.pbio.3000958 (PMC7644102; doi:10.1371/journal.pbio.3000958)
Supplement: Attachment — Submitted filename: Response_to_referees.pdf [file pbio.3000958.s007.pdf]

We thank the reviewers for reading our manuscript and providing constructive input. Below we have addressed the comments point by point and believe that the manuscript has improved with the suggested changes.

Reviewer #1: [identifies himself as Alexey Amunts]

The cryo-EM study by Ehrenbolger and coworkers presents a single-particle structural model of hibernating ribosome from microsporidium pathogen family organism Paranosema locustae in complex with a recycling factor Lso2. Although microsporidian ribosomes generally have reduced rRNA, correlated with minimal known eukaryotic DNA, here the authors have chosen species with a less reduced variant, and remarkably identified an expansion segment reduced to a single nucleotide. From the high-resolution structural models, the authors assigned a function for this nucleotide and could explain why it retained in evolution. The central aspect of the work is the factor Lso2 that blocks catalytic sites.

The technical work is well executed, the analysis conducted with attention to details and well presented. The comparison to other characterised and more familiar ribosomal complexes is interesting and helpful.

We thank Dr. Amunts for the assessment of our work and his positive comments.

I only have minor comments:

- Figure 4 and related text: perhaps indicate the local resolution for the nucleotide density to assure readers.

As proposed, we included the local resolution estimation for the nucleotide binding region (eL20, uL6) in supplemental figure 2 panel D:

D

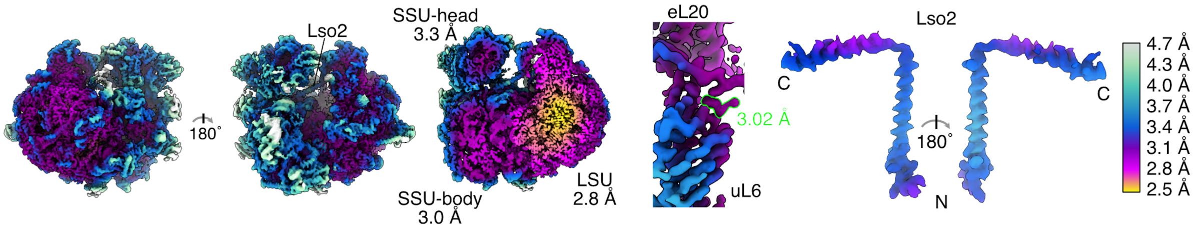

In addition, we reference this figure at appropriate locations and have added the information to the main text on line 112ff:

"In addition, we discovered density for a nucleotide with a local resolution of 3 Å in the interface between uL6 and eL20 (**Fig 1, S2 Fig**), acting as a remnant of a removed expansion segment."

- Methods: clarify why three P. locustae genomes were needed.

We appreciate the reviewer pointing out the possible misunderstanding regarding the *P. locustae* genome database. During the time this study was performed, no single complete, curated and fully annotated genome was available for *P. locustae*. We therefore used the three available, non-curated and partially annotated, *P. locustae* genomes to ensure all ribosomal protein and rRNA sequences present

could be identified. In August 2020, the currently most complete genome was published by Chen et. al (PMID: 32783805), which has not changed our findings. To clarify any potential misunderstanding, we have further elaborated on this aspect in the methods section on line 344ff:

"At the start of this study, no complete and annotated genome was available for *P. locustae*. Hence, to ensure complete coverage of all the relevant ribosomal protein and RNA sequences, we used three available, but non-annotated, *P. locustae* genomes [44–46] to create a local database. This database was used to identify *P. locustae* ribosomal protein and rRNA sequences (**S2 Table**)."

Reviewer #2:

This manuscript by Ehrenbolger and colleagues replicates a previously published study by Beckmann's group in which Wells and colleagues described hibernation and reactivation of 80S ribosomes with protein Lso2. Because PLoS Biology publishes "scooped" work as a means of improving reproducibility, I request the authors to replicate the most exciting and key finding of Wells and colleagues' study: their finding that Lso2 is not simply an additional hibernation factor, but a factor that helps reactivate hibernating ribosomes by predisposing them for Dom34-mediated splitting into subunits. Otherwise, I find the study by Ehrenbolger and colleagues too incremental and superficial to warrant its publication in PLoS Biology.

Specifically, I request the authors to express microsporidian homologs of Dom34-recycling system and repeat the in vitro splitting assay shown in Figure 5 of Beckmann's group study (https://journals.plos.org/plosbiology/article?id=10.1371/journal.pbio.3000780).

We thank reviewer #2 for the comments and suggestions. The study noted by the reviewer and the outlined ribosome splitting and reactivation assay could give insights into how the microsporidian ribosome is rapidly reactivated post-infection. While we believe this recommendation is worth pursuing, specific challenges significantly increase the complexity of this analysis:

1. There is no characterization of the recycling pathways and their constituent proteins in microsporidia. A BLAST search of the *P. locustae* genome using the *S. cerevisiae* Dom34 sequence allows us to identify a single potential pelA-domain containing protein; however, it shares only 20% sequence identity with yeast Dom34. A comprehensive analysis would therefore be required to verify that this protein is indeed the microsporidian Dom34 homolog.

2. Although isolation of ribosomes from microsporidia is relatively straight-forward, the cultivation and isolation of spores from migratory locusts requires a 2-month development cycle (see methods). This long delay (before even actually performing the splitting assay) may negatively impact the temporal significance of our study.

For these reasons, we believe that pursuing a Dom34 splitting-assay is outside of the scope of this work and the PLOS Biology 'Short Report' format.

Also, to extend their analysis, the authors could possibly speculate in their Discussion about why Lso2 does not bind ribosomes during stages of active growth? Does Lso2 possess any

serine/threonine residues (at the rRNA/protein interface) which could undergo phosphorylation in response to growth stimuli?

Based on known information about the bacterial hibernation factor RMF (PMID: 30476446 and internal citations), which is quickly degraded at both the protein and transcript level in nutrient-rich conditions, we might speculate that Lso2 is rapidly degraded post-infection. Unfortunately, there is little proteomic or transcriptomic data currently available for microsporidia comparing different lifecycle stages, and the only study containing any insights into Lso2 (PMID: 26837419), compares only the pre-germination and post-germination (but still extracellular) stages. In this work, no significant difference at the transcript level was found. It is important to note that we would expect further transcriptional changes to occur once the sporoplasm is injected into host cells.

The suggestion that regulation occurs via post-translational modifications caused us to reconsider the residues at the binding interface. Indeed, the N-terminal protein-rRNA interface does contain two S/T residues that may act as a potential regulatory switch of this interaction. We prefer not to speculate based solely on the presence of phosphorylatable residues, especially since microsporidian genomes are significantly depleted in kinases (as few as 31 predicted proteins, PMID: 25549259); however, we have included sentences outlining possible regulatory mechanisms on line 271ff:

"Previous analyses indicate that *mdf1* transcription is increased towards the end of sporulation [9], suggesting Mdf1 activity is controlled by regulating protein concentration. Although no similar data are available for Lso2 in microsporidia, other hibernation factors are rapidly degraded upon return to nutrient-rich conditions [34,35], suggesting temporal regulation is common. Alternatively, Lso2 interactions with ribosomes may be mediated by post-translational methylations to the numerous lysine residues present throughout the protein, or by phosphorylation of the relatively conserved serine and threonine residues at the N-terminal protein-rRNA binding interface (**Fig 2, S3 Fig**). Further work is needed to segregate the functional roles for various hibernation factors, and identify the mechanisms by which hibernation factors are regulated."

As a minor comment, I disagree with the authors's statement that their study highlights "diversification of the microsporidian ribosome". In my opinion, their study highlights diversification and redundancy of ribosome hibernation mechanisms. For instance, yeast S. cerevisiae can hibernate their 80S ribosomes by using either protein Lso2 or Myb protein 1. But it does not mean that S. cerevisiae 80S ribosomes have two distinct and diversified populations for each of these hibernation factors.

The reviewer is correct to note that this study highlights both diversification and redundancy of hibernation mechanisms. However, one of the additional findings that we note and expand upon in our paper is the variation in evolutionary compaction of the ribosome between different microsporidian species. This is particularly visible in figure 3, where differences in both the size of expansion segments and the protein content (msL1) between the two microsporidian ribosomes are apparent. In this regard, the title highlights the diversification of microsporidian ribosome contents compared to one another.

Although both *V. necatrix* and *P. locustae* ribosomes were isolated in a similar manner, their ribosomes were bound with high occupancy by different hibernation factors. This could suggest that different species of microsporidia may have developed preferences for the utilization of alternative hibernation factors, for similar functions. In this way, we believe that our work highlights the diversification of the utilization of hibernation factors in microsporidian ribosomes.

We hope that this explanation clarifies our thought process for titling our work 'Differences in structure and hibernation mechanism highlight diversification of the microsporidian ribosome,' and appreciate the consideration the reviewer put into the meaning of our work.

Overall, this study is stunningly illustrated and written in a very articulate and simple manner, and I am sure the authors will be able to easily address my comments.

We are delighted to hear that the reviewer appreciated our efforts to make a comprehensible and visually pleasing paper!

Reviewer #3:

This manuscript reports the structure of an inactive microsporidian (P. locustae) ribosome bound by the ribosome hibernation factor Lso2. The ribosome structure presented here shows an intermediate level of rRNA reduction relative to the recently published V. necatrix ribosome. The Lso2 hibernation factor also contrasts with the MDF1 and MDF2 hibernation factors in the V. necatrix ribosome, and allows direct comparison with Lso2 / CCDC124 in other eukaryotes. It is also reported that the structure contains a bound nucleotide between uL6 and uL20. This nucleotide is interpreted to serve as a replacement for an eukaryotic expansion segment that has been reduced in P. locustae.

The structure of this microsporidian ribosome contributes to our understanding of the translational machinery, based on its phylogenetic position and reduced ribosome size. The Lso2 structure is likewise valuable and is complemented by functional studies in yeast (Wang et al., 2018). However, it seems that the presence of the bound nucleotide is interpreted quite strongly in the absence of corroborating data. The proposed model is plausible, but the nucleotide might bind adventitiously, or only in the hibernating state. Gathering the sort of genetic or biochemical evidence that could support this claim is beyond the scope of revisions. However, it is important that the discussion of this structural observation acknowledge these limitations.

We thank the reviewer for their accurate summary of our work, and for raising this important concern. We have adjusted the descriptions of the importance of the bound nucleotide throughout the text to avoid overstating our conclusions, and have changed the discussion to stress the limitations of our model:

Line 43ff

"In one case, the near complete reduction of an expansion segment has resulted in a single bound nucleotide, which **may** act as an architectural co-factor to stabilize a protein-protein interface"

Line 84ff

"A single structural nucleotide, discovered at the interface of two ribosomal proteins, serves as the remaining element of a removed rRNA segment and **may** act as the most minimal version of an ES."

Line 236ff:

"Although the high conservation of this binding site in eukaryotes suggests an important and conserved function, it is possible that this interaction is a result of proximity and opportunity. Further

studies, such as the deletion of this nucleotide from yeast ES39, would be necessary to verify the functional significance of this interaction."
